# Astrocyte Stellation, a Process Dependent on Rac1 Is Sustained by the Regulated Exocytosis of Enlargeosomes

**DOI:** 10.1002/glia.22280

**Published:** 2011-12-05

**Authors:** Gabriella Racchetti, Rosalba D'Alessandro, Jacopo Meldolesi

**Affiliations:** 1 Scientific Institute San Raffaele, Division of Neuroscience and IIT Network, Research Unit of Molecular Neurosciencevia Olgettina 58, Milan, Italy; 2 S. De Bellis Scientific InstituteCastellana Grotte, Bari Italy

**Keywords:** small GTPases, cytoskeleton, surface expansion, membrane traffic, process outgrowth

## Abstract

Cultured astrocytes exhibit a flat/epitelioid phenotype much different from the star-like phenotype of tissue astrocytes. Upon exposure to treatments that affect the small GTPase Rho and/or its effector ROCK, however, flat astrocytes undergo stellation, with restructuring of cytoskeleton and outgrowth of processes with lamellipodia, assuming a phenotype closer to that exhibited *in situ*. The mechanisms of this change are known only in part. Using the ROCK blocker drug Y27632, which induces rapid (tens of min), dose-dependent and reversible stellations, we focused on two specific aspects of the process: its dependence on small GTPases and the large surface expansion of the cells. Contrary to previous reports, we found stellation to be governed by the small G protein Rac1, up to disappearance of the process when Rac1 was downregulated or blocked by a specific drug. In contrast cdc42, the other G-protein often involved in phenotype changes, appeared not involved. The surface expansion concomitant to cytoskeleton restructuring, also dependent on Rac1, was found to be at least partially sustained by the exocytosis of enlargeosomes, small vesicles distinct from classical cell organelles, which are abundant in astrocytes. Exhaustion of stellation induced by repeated administrations of Y27632 correlated with the decrease of the enlargeosome pool. A whole-cell process like stellation of cultured astrocytes might be irrelevant in the brain tissue. However, local restructuring of the cytoskeleton coordinate with surface expansion, occurring at critical cell sites and sustained by mechanisms analogous to those of stellation, might be of importance in both astrocyte physiology and pathology. © 2011 Wiley Periodicals, Inc.

## INTRODUCTION

The profound structural differences between the flat, epithelioid astrocytes cultured *in vitro* in serum-containing media, and the classical *in-vivo* star-like phenotype, with multiple ramified processes covered with filopodia and lamellipodia, is known since 40 years. Soon thereafter, cultured astrocytes were found to reacquire at least part of their *in situ* phenotype, with reduction of the cell body and outgrowth of processes, when exposed to either dibutyryl cAMP (db-cAMP) or brain extracts in serum-free medium (Lin et al., 1973; Lin and Mitsunobu, 1974; Moonen et al., 1975). The process, often named stellation, was shown to be induced also by other agents: cGMP, administered as the 8-bromo analogue (8Br-cGMP) or generated in response to the atrial natriuretic peptide (Boran and Garcia, 2007); AICAR, an activator of the AMP-dependent protein kinase (Favero and Mandel, 2007); interleukin 1β (John et al., 2004); ATP (Neary et al., 1994) acting via activation of the adenosine A2 receptor (Rosso et al., 2007); various nonsteroidal anti-inflammatory agents (Lichtenstein et al., 2010); botulinum toxin C3 (Suidan et al., 1997). Conversely, flattening of stellate astrocytes was induced not only by serum but also by thrombin and lysophosphatidic acid (Holtje et al., 2005; Suidan et al., 1997).

Many aforementioned treatments were shown to operate by affecting the small GTPase RhoA or its effector kinase, ROCK (Favero and Mandel, 2007; John et al, 2005; Lichtenstein et al., 2010; Salhia et al., 2005; Suidan et al., 1997). High Rho and ROCK activities are necessary for flattening; repression of RhoA (Boran and Garcia, 2007; Holtje et al., 2005; John et al., 2005; Rosso et al., 2007) or blockade of ROCK (Abe and Misawa, 2003; John et al., 2005; Lau et al., 2011; Salhia et al., 2005) induce the conversion of flat to stellate astrocytes. The expansion or marked reduction of the cell body accompanied by the disappearance or outgrowth of branched processes, respectively, are sustained by the reshaping of the cytoskeleton, a well-known target of Rho GTPases (Hall, 2005; Ridley, 2006). Specifically, the network of thin actin filaments of astrocytes *in situ* is converted in culture into a system of robust stress fibres impinging onto and running below the plasma membrane. During stellation these fibers collapse and are converted back to the filamentous network (Favero and Mandel, 2007; John et al., 2005; Salhia et al., 2005).

In this study, carried out by using the rapidly effective ROCK blocker Y27632 (Abe and Misawa, 2003; John et al., 2005; Lau etal., 2011; Salhia et al., 2005), we investigated two specific issues. The first is whether stellation and flattening depend on Rac1, a small GTPase of the Rho family constitutively repressed by ROCK (Nakayama et al., 2008; Ohta et al., 2006; Takefuji et al., 2007; Yamaguchi et al., 2001). Rac 1 is known to govern dendritic outgrowth in various types of neural cells (Hirose et al., 1998; Racchetti et al., 2010). In astrocytes, studies had been carried out, however with conflicting results (John et al., 2005; Lichtenstein et al. 2010; Rosso et al., 2007; Salhia et al., 2005; Suidan et al., 1997). The second issue concerns the nature and trafficking of the membranes necessary for stellation and flattening to occur. The surface area of flat astrocytes is much smaller than that of stellate astrocytes. Conversion of one phenotype to the other requires therefore the addition or removal of membrane to and from the plasmalemma (Chieregatti and Meldolesi, 2005; Morris and Homan, 2001). This traffic could involve processes such as regulated exocytosis (Chieregatti and Meldolesi, 2005), endocytosis (Maxfield and McGraw, 2004) and vesicle shedding to the extracellular space (Cocucci etal., 2007), never investigated in astrocytes in relation to flattening and stellation.

## MATERIALS AND METHODS

The mouse anti-Ahnak monoclonal antibody (mAb) was developed in our laboratory (Borgonovo et al., 2002). Secramine was the gift of T. Kirchhausen. Other Abs and chemicals were obtained from the following sources: mouse anti-β-tubulin mAb, rabbit anti-α actin plyclonal Ab (pAb), DAPI, nocodazol, 8Br-cGMP, db-cAMP, ionomycin, latrunculin A, wortmannin: Sigma-Aldrich; Y27632 and ionomycin: Calbiochem; rabbit anti-GFAP pAb: Dako Cytomation; mouse anti-58K mAb: Abcam; rabbit anti-calnexin pAb: Stressgen Biotech; mouse anti-TGN38 mAb: Thermo Fischer Scientific; guinea-pig anti vGLUT1 and anti-vGLUT2 pAbs: Chemicon; anti-EEA2 pAb: Santa Cruz; anti-GLAST mAb: Myltenyl Biotec; mouse anti-ezrin mAb, FITC-conjugated, TRITC-conjugated and far-red conjugated goat anti-mouse pAb, goat anti-rabbit pAb, FITC-conjugated phalloidin and Lipofectamine 2000: Invitrogen; EHT 1864: Tocris. The GFP-plasmids with scrambled or Rac1-specific shRNA were from Origene.

### Cell Culture

Primary astrocytes were obtained from the cortex of P2 Sprague-Dawley rat pups (Charles River), sacrificed according an approved procedure (Prada et al, 2011). Freshly dissected cortices were disaggregated, washed, and seeded. After 8 days, they were depleted of microglia by shaking and seeded again for 2 days (Prada et al., 2011). wtPC12, PC12-27, and SH-SY5Y cells were as in Racchetti et al. (2010). Incubation medium was MEM with 10% horse serum, reduced to 1% in the starving medium.

### Cell Processing, Immunofluorescence Microscopy, and Micrometry

Cultured cells were fixed in either Cytoskelfix (Cytoskeleton) (4 min at −20°C) or 4% formaldehyde in phosphate buffer (10 min, 4°C), and then washed. For transient Rac1 downregulation, the cells, seeded on slides coated with polylysine, were exposed for 48 h to the shRNA (Tomasoni et al., 2001), scrambled or specific. The latter, chosen out of four based on efficacy, was as follows: CGAGGACTCAAGACAGTGTTTGATGAAGC. Cells were then stimulated with Y27632 for 1 h and fixed in 4% formaldehyde. Processesoutgrown during treatment were defined so when thin (<1 μm) and at least 5 μm long. Tissue slices 30 μm thick, obtained from the brain cortex of P2 rats, were fixed and processed as described by Prada et al. (2011). Whole cell and surface immunolabelings were carried out in cells permeabilized or not with 0.4% saponin before the first antibody. Subsequent steps and recordings were as in Racchetti et al. (2010). Quantitation of fluorescence signals in either permeabilized or nonpermeabilized cells was made using the IMAGEJ program (Rasband WS, ImageJ, U.S. NIH,Bethesda, MD, http://rsb.info.nih.gov/ij/, 1997–2011). Values given are averages ± SD. The number and length of major processes were estimated using the IMAGEJ program in at least 25, Y27632-treated astrocytes for each time-point, and then subtracted of the average baseline estimated in astrocytes transfected with the scrambled shRNA.

### Spinning Disc Time-Lapse Imaging

Spinning disc time-lapse imaging was performed at the IFOM-IEO campus (Milan). Astrocytes, transfected with either the scrambled or the Rac1-specific shRNAs, were plated at low confluence on 35-mm glass-bottomed dishes (MatTek) coated with poly-l-lysine (100 μg/mL), then starved overnight and finally treated with Y27632 in a Nikon Ti inverted microscope (Nikon) equipped with a Perkin Elmer Ultraview VoX spinning disc confocal system (Perkin Elmer) controlled by Volocity software (Perkin Elmer). Image sequences were converted into avi-files with the IMAGEJ program.

### Western Blotting

Western blottings were carried out and quantized as described by D'Alessandro et al. (2008).

## RESULTS

### Rapid Stellation Induced by Y27632

**Fig. 1:**
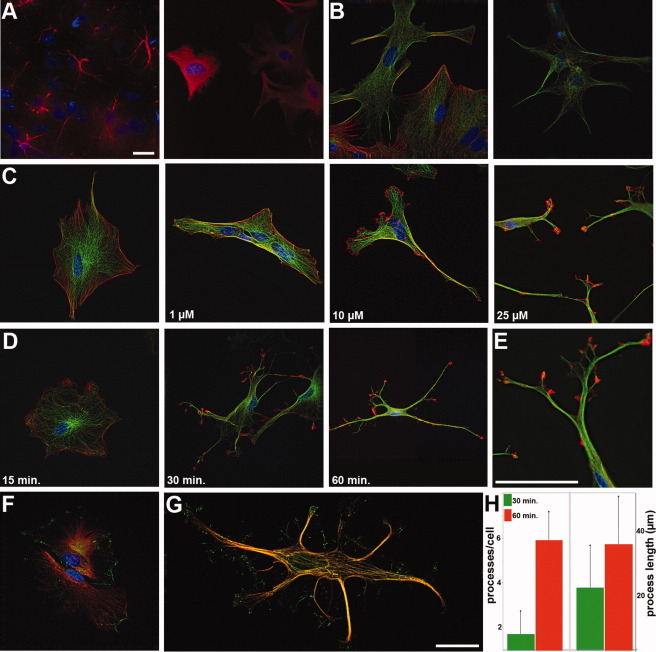
Astrocyte stellation induced by 8Br-cGMP and Y27632. (**A**) Astrocytes of the brain tissue (left) and cultured *in vitro* (right) immunolabeled for GFAP. The cells in B-E were dually immunolabeled for β-tubulin (green) and α-actin (red). In the microscopy panels of this and the following figures nuclei were labeled blue by DAPI. (**B**) astrocytes preincubated in complete medium (left) or pre-starved overnight in low (1%) serum medium (right) were treated with 8Br-cGMP (100 μM, 16 h). (**C**–**E**) Dose dependence and time-course of the phenotype changes induced by treatment with Y-27632 in astrocytes. In (C), from left to right, 1 h treatment with 0, 1, 10, and 25 μM Y27632; in (D), from left to right, 25 μM Y27632 administered for 15, 30, and 60 min. (E) Enlargement of a 60 min-treated cells showing lamellipodia emerging from processes. (**F**, **G**) Distribution of ezrin and GFAP in astrocytes incubated for 0 and 60 min with 25 μM Y27632. Bars in (A) (valid in B–D and F), (E), and (G) are 10 μm. (**H**) Number/cell and length of the major processes induced in populations of at least 25 astrocytes by 30 and 60 min treatment with 25 μM Y27632.

The two panels of Fig. 1A illustrate the astrocyte phenotypes used for references of our studies: the star-like of the rat brain cortex tissue (left) and the flat, epithelioid astrocytes, dissociated from brain cortex of P2 rats and cultured for 10 days in complete, 10% serum-containing medium (right). Fig. 1B compares the changes induced by one of the agents previously reported to induce stellation, 8Br-cGMP (100 μM, 16 h), used here for reference. 8Br-cGMP was administered to flat astrocytes cultured in the standard medium (left) or prestarved overnight in the medium containing 1% horse serum (right).

Inthe first case, many astrocytes maintained their flat phenotype with robust stress fibers and only a few short, thick expansions; in the second stellation appeared more advanced, with no appreciable stress fibers. Upon shorter treatments with 8Br-cGMP the changes were less pronounced, similar to those induced by db-cAMP administered for various periods of time to prestarved cells (not shown).

When induced by Y27632, stellation was in contrast fast and extensive, similar in both complete and low-serum media. Upon 1 h treatment (Fig. 1C), 1 μM drug concentration already induced changes of the astrocyte shape from flat to elongate, with reorganization of the cytoskeleton and loss of most centrifugal stress fibers. A more advanced change, with decreased cell body area and appearance of multiple globular surface sprouts enriched of α-actin together with few long processes, was induced by 10 μM drug. Twenty-five micromolars yielded fully stellate astrocytes, with a widespread network of α-actin concentrated at the tips of numerous, long, thin, often branched processes (Fig. 1C). The time-course of these effects was also investigated (Fig. 1D). A partial reorganization of the cytoskeleton in the flat cell body, with generation of globular, α-actin-enriched surface sprouts, was evident at 15 min; appearance of typical processes enriched of α-actin at 30 min. At 1 h, the cells exhibited very small bodies and many thin processes covered by lamellipodia containing α-actin (see the high power Fig. 1E). The number and length of the processes grown in stellating astrocytes during 60 min treatment with 25 μM Y27632 (Fig. 1H) were analogous of those of brain cortex astrocytes (Chao et al., 2002). We conclude that the analogy of the Y27632-stellated astrocytes with the star-like astrocytes of the tissue is considerable, suggesting mechanistic commonalities in their establishment.

Stellation induced also changes in the distribution of the actin-binding protein ezrin. In flat astrocytes, ezrin was spread in the cytoplasm together with GFAP (Fig. 1F). Upon 1 h treatment with 25 μM Y27632, ezrin was redistributed in part to the processes up to lamellipodia from which GFAP was excluded (Fig. 1G). This property, already described in tissue and cultured astrocytes by Derouiche and Frotscher (2001), appears of functional importance (Lavialle et al., 2011).

### Role of Microtubules and Microfilaments

The cytoskeleton is known as a key player in the flattening/stellation processes (Favero and Mandel, 2007; John etal., 2005; Salhia et al., 2005). Details about the role of microtubules and microfilaments were however still undefined. Cultured astrocytes were therefore pretreated with either nocodazol, that induces depolymerization of microtubules, or latrunculin A, that sequesters α-actin monomers and therefore perturbs the kinetics of microfilaments. The effects of these treatments were investigated *per se* and in relation to the stellation induced by 25 μM Y27632 applied together with the two cytoskeleton-addressed drugs.

**Fig. 2:**
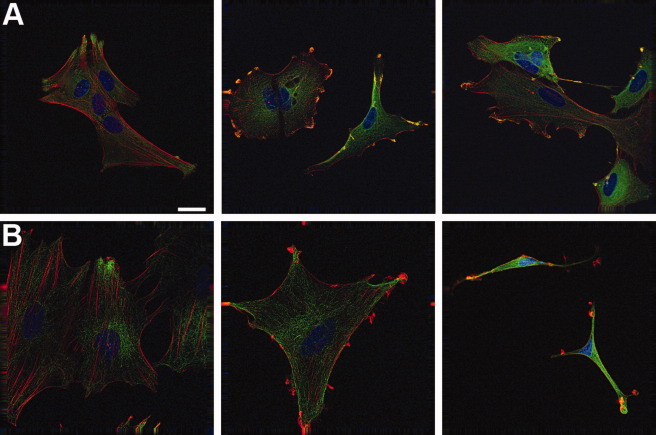
Effects of nocodazol and latrunculin A on cultured astrocytes treated or not with Y27632. The left panels of (**A**) and (**B**) show cells treated with nocodazol (A, 30 μM, 1 h) and latrunculin A (B, 5 μM, 15 min). The central and right panels show cells that, upon treatment with nocodazol (A) and latrunculin A (B) were further incubated with Y27632 (25 μM, 60 min) together with the cytoskeleton-addressed drugs. All cells were immunolabeled for β-tubulin (green) and α-actin (red). Bar in (A), valid in (B), is 10 μm.

In flat astrocytes, nocodazol pretreatment (30 μM, 1 h) induced only minor changes. The drug, however, prevented most changes induced by Y27632, that did not go beyond the redistribution of α-actin with appearance of multiple enriched surface sprouts. Outgrowth was limited to a few, thick expansions. Thus, true stellation did not take place (Fig. 2A). The latrunculin A pretreatment (5 μM, 15 min) failed to affect markedly the phenotype of flat astrocytes, including stress fibers. It did however change the effects of Y27632. The shape of latrunculin A/Y27632-treated cells was variable, ranging from flat to stellate. α-Actin distribution was disordered, with formation of scattered clusters protruding from the cell surface (Fig. 2B), while the processes exhibited only few or no surface lamellipodia. Both cytoskeleton-addressed drugs affect therefore stellation, however in different ways. Nocodazol blocks process outgrowth; latrunculin A precludes a correct reorganization of the actin network.

### Stellation Induced by Y27632 Depends on Rac1

**Fig. 3:**
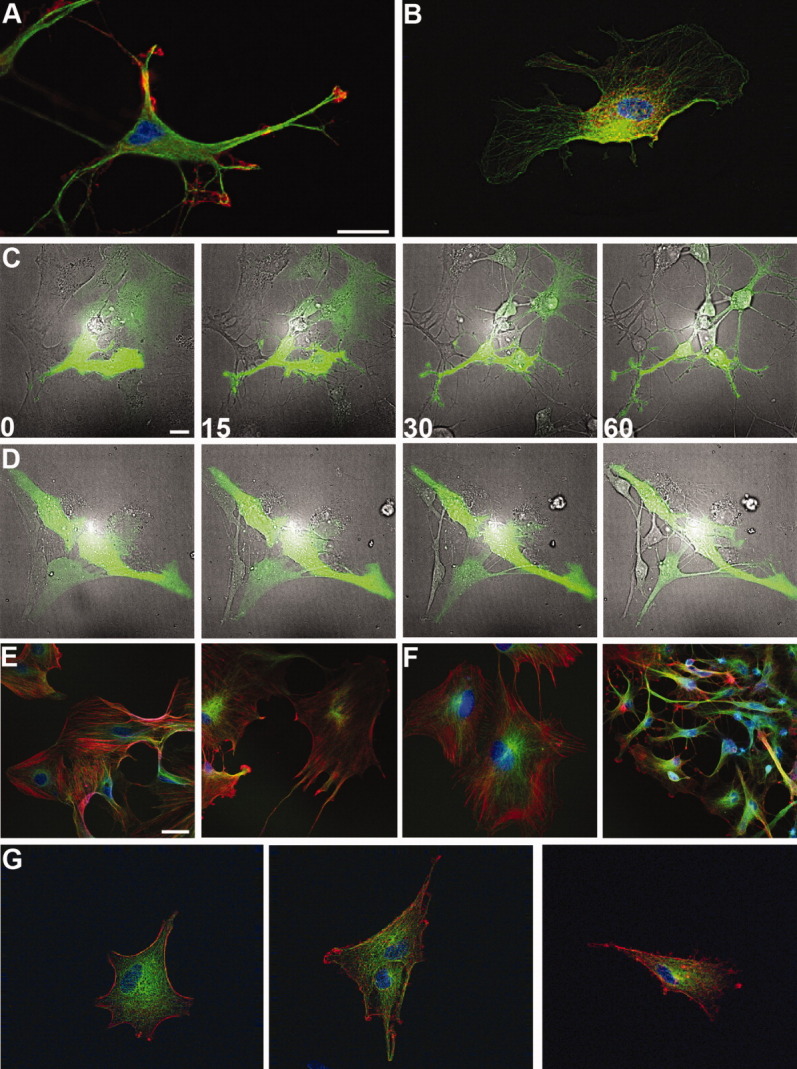
Astrocyte stellation depends on Rac1. (**A**, **B**) Cultured astrocytes 48 hr after transient transfection of shRNA, scrambled (A) or Rac1-specific (B), followed by treatment with Y27632 (25 μM, 60 min). (**C**, D) Subsequent frames of astrocytes transfected (GFP-positive, green) or not (GFP-negative) with the scrambled (C) or the Rac1-specific (D) shRNA, analyzed by spinning disc time-lapse imaging upon administration of Y27632 (25 μM). The numbers on the frames of C (valid also for D) mark the 4 min upon addition of the drug. (**E**–**G**) Astrocytes preincubated with the specific blocker of Rac1, EHT 1864 (5 μM, overnight, E), the specific blocker of cdc42 secramine (dissolved in 1% DMSO-0.5% BSA; 15 μM, 1 h, F) or the PI3 kinase blocker, wortmannin (0.3 μM, 1 h, G), followed by 1 h incubation with the same drugs without (left) or with (right; in G also the middle panel) Y27632 (25 μM). The cells of (A), (B), and (E–G) were immunolabeled for β-tubulin (green) and α-actin (red). Bars in (A) (valid in B), (C) (valid in D), and (E) (valid in F, G) are10 μm.

Various types of experiments were carried out to investigate the possible Rac1-dependence of stellation. Cultured astrocytes transfected with either the scrambled or the Rac1-specific shRNAs positive for GFP were treated with Y27632 (25 μM, 1 h) 48 h later. Most cells transfected with the scrambled shRNA exhibited the typical, Y27632-induced stellate phenotype (compare Fig. 3A to Fig. 1C,D, right panels), with outgrowth of multiple thin and branched processes. In contrast, in the cells transfected with the Rac1-specific shRNAs the changes induced by Y27632 were attenuated or completely absent (Fig. 3B).

shRNA-transfected astrocytes were also analyzed by spinning disc time-lapse imaging upon addition of Y27632. A comparison of the scrambled shRNA-transfected cells, positive for GFP, with the GFP-negative, nontransfected cells of the same populations, revealed no differences, i.e. the GFP-positive and negative cells developed concomitantly the typical stellate phenotype induced by the drug (Fig. 3C; whole recording in Supp. Info. Fig. 1). In contrast, in the dishes transfected with the Rac1-specific shRNA the GFP-positive cells did not modify significantly their shape, whereas the GFP-negative cells underwent full stellation responses (Fig. 3D and Supp. Info. Fig. 2).

The role of Rac1 and that of cdc42, another GTPase of the Rho family involved in the regulation of the cytoskeleton (Hall, 2005; Ridley, 2006), was further investigated by a pharmacological approach. Cultured astrocytes exposed overnight to EHT 1864 (5 μM), a Rac1 blocker with no effect on cdc42 (Shutes et al., 2007), exhibited only minor changes of phenotype, and no stellation, when exposed to Y27632 (Fig. 3E). In contrast, most astrocytes treated with secramine (15 μM, 1 h), a blocker of cdc42 inactive on Rac1 (Pelish et al., 2006), exhibited Y27632-induced stellations similar to those of control cells (Fig. 3F).

Previous evidence in a variety of cell types (reviewed by Fukata et al., 2003) demonstrated the effects of Rac1 on the cytoskeleton to depend on its cooperation with PI3 kinase. We therefore investigated whether the Y27632-induced stellation was affected by wortmannin, a blocker of the enzyme. Figure 3G shows that wortmannin pretreatment (0.5 μM, 1 h) induced some alteration of the cytoskeleton with attenuation of the robust α-actin stress fibers. The subsequent application of Y27632 failed to induce any significant stellation. Both the shRNA and the pharmacological results confirmed therefore that the stellation effect of Y27632 depends on the activation of Rac1, possibly working co-ordinately with PI3 kinase.

### The Astrocyte Plasma Membrane Expansion During Y27632-induced Stellation Is Sustained by the Exocytosis of Enlargeosomes

By analogy to neurons and neurosecretory cells (Pfenninger, 2009; Meldolesi, 2011), the astrocyte vesicles sustaining membrane expansion and process outgrowth induced by Y27632 were expected to be specific, distinct from the clear and dense-core vesicles competent for the regulated discharge of gliotransmitters (reviewed by Parpura and Zorec, 2010; Volterra and Meldolesi, 2005). In various types of neural cells (PC12, SH-SY5Y, immature neurons) neurite outgrowth triggered by Y27632 had been shown to be sustained by the exocytosis of enlargeosome (Racchetti et al., 2010), a vesicle known to be present in cultured astrocytes (Borgonovo et al., 2002). So far, however, the exocytosis and function of enlargeosomes in astrocytes had never been investigated.

**Fig. 4:**
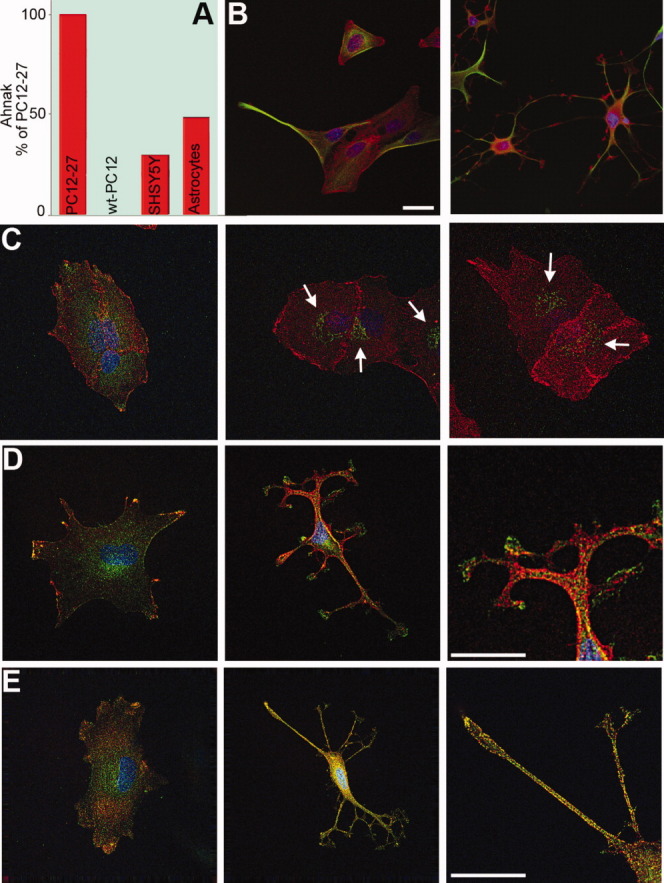
Enlargeosomes participate in the surface enlargement of stellation. (**A**) Western blot results showing that astrocytes are rich of enlargeosomes (marker Ahnak, expressed in the range of enlargeosome-rich neural cells, PC12-27 and SH-SY5Y). (**B**) Astrocytes before (left) and after (right) stellation, immunolabeled for GFAP (green) and Ahnak (red). (**C**) Flat cultured astrocytes dually labeled for Ahnak (red) and markers of cytoplasmic organelles (green): calnexin for endoplasmic reticulum (left panel), 58 K for Golgi complex (arrows, middle panel), TGN38 for transGolgi network (arrows, right panel). (**D**, **E**) astrocytes before (left panels) and after Y27632-induced (25 μM, 60 min) stellation (middle panels) dually immuno-labeled for Ahnak (red) and the vesicular glutamate transporters vGLUT1 (D) and vGLUT2 (E) (green). The right panels of (D, E) are higher magnification, deconvolved images of the corresponding middle panels. Bars in (B) (valid in C and left/middle panels of D, E), and in (D, E) right panels, are 10 μm.

Western blot results in cultured, flat astrocytes revealed the expression of the enlargeosome marker, Ahnak, to be much higher than in wtPC12 and in the same range as other cells rich of enlargeosomes, PC12-27 and SH-SY5Y (Fig. 4A; see Prada et al., 2011). Ahnak immunolabeling showed the marker concentrated in numerous small puncta spread throughout the cytoplasm (Fig. 4B, left panel), that upon stellation redistributed also to the processes (Fig. 4B, right panel). As in many other cell types, the Ahnak-positive puncta did not colocalize with markers of classical organelles such as the ER, Golgi complex, and transGolgi network (calnexin, 58K, TGN38, respectively, Fig. 4C), concentrated in discrete areas of the cytoplasm. Likewise, Ahnak did not colocalize with various glutamate transporters, neither the clear vesicle vGLUT1 (Fig. 4D) nor the plasma membrane transporters GLAST and EAA2, which in cultured astrocytes exhibited a widespread cytoplasmic distribution (not shown). A partial colocalization of Ahnak was observed only with the vesicular glutamate transporter vGLUT2. Deconvolution analyses of the samples (Fig. 4E) revealed it to occur in discrete puncta distributed in the cell body and the processes, especially proximal to the surface. The relevance of this colocalization remains to be investigated.

**Fig. 5:**
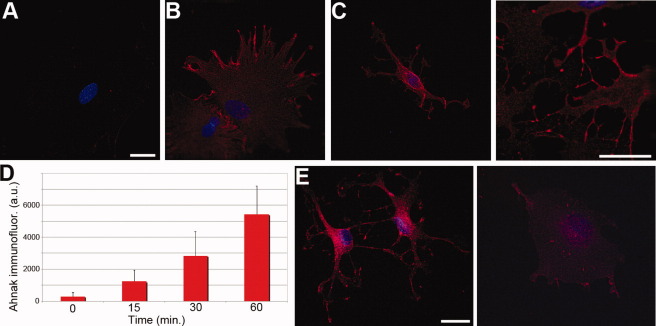
Enlargeosome exocytosis induced by Y27632 depends on Rac1. (**A**) A resting flat astrocyte surface-negative for the enlargeosome marker, Ahnak. (**B**) Ahnak surface-labeling in astrocyte stimulated with ionomycin (1μM, 10 min). (**C**) Surface Ahnak labeling in astrocytes stellated by Y27632 (25 μM, 1 h). (**D**) Quantitative immuno-labeling of surface Ahnak labelling in groups of at least 25 cells, treated with Y27632 (25 μM) for the indicated times. (**E**) Ahnak surface labelling in astrocytes transfected (see Fig. 3) with the scrambled (left) or the Rac1-specific (right) shRNA, and then treated with Y27632 (25 μM, 1 h). Bars in (A), valid for (B, C) left panel; (C) right panel and (E) are 10 μm. [Color figure can be viewed in the online issue, which is available at wileyonlinelibrary.com.]

Proof of the involvement of enlargeosomes in stellation was obtained by surface immunolabeling of nonpermeabilized astrocytes. As expected in view of the lumenal localization of Ahnak, its surface immunolabeling of resting astrocytes was negative (Fig. 5A). Treatment with the Ca^2+^ ionophore ionomycin (1 μM, 10 min: Borgonovo et al., 2002), induced the surface appearance of the marker accompanied by an expansion of the plasma membrane (Fig. 5A). The phenotype of the ionomycin-treated cells, however, was profoundly different from star-like. The cells remained flat, with thick protrusions much different from the thin processes of stellate cells (Fig. 5B). Upon treatment with Y27632, in contrast, the surface immunolabeling of Ahnak appeared concomitant with stellation (compare Fig. 5D to Fig. 1H), distributed at both the cell body and the processes, up to lamellipodia (Fig. 5C). It was unaffected in the cells transfected with the scrambled shRNA, and largely blocked in those transfected with the shRNA specific for Rac1, which kept their flat/epithelioid phenotype unaltered (Fig. 5E). Taken together these results demonstrate that stellation induced in astrocytes by Y27632 occurs together with strong enlargeosome exocytic responses, also dependent on Rac1.

### Repetitive Applications of Y27632 Lead to Exhaustion of the Stellation Responses

**Fig. 6:**
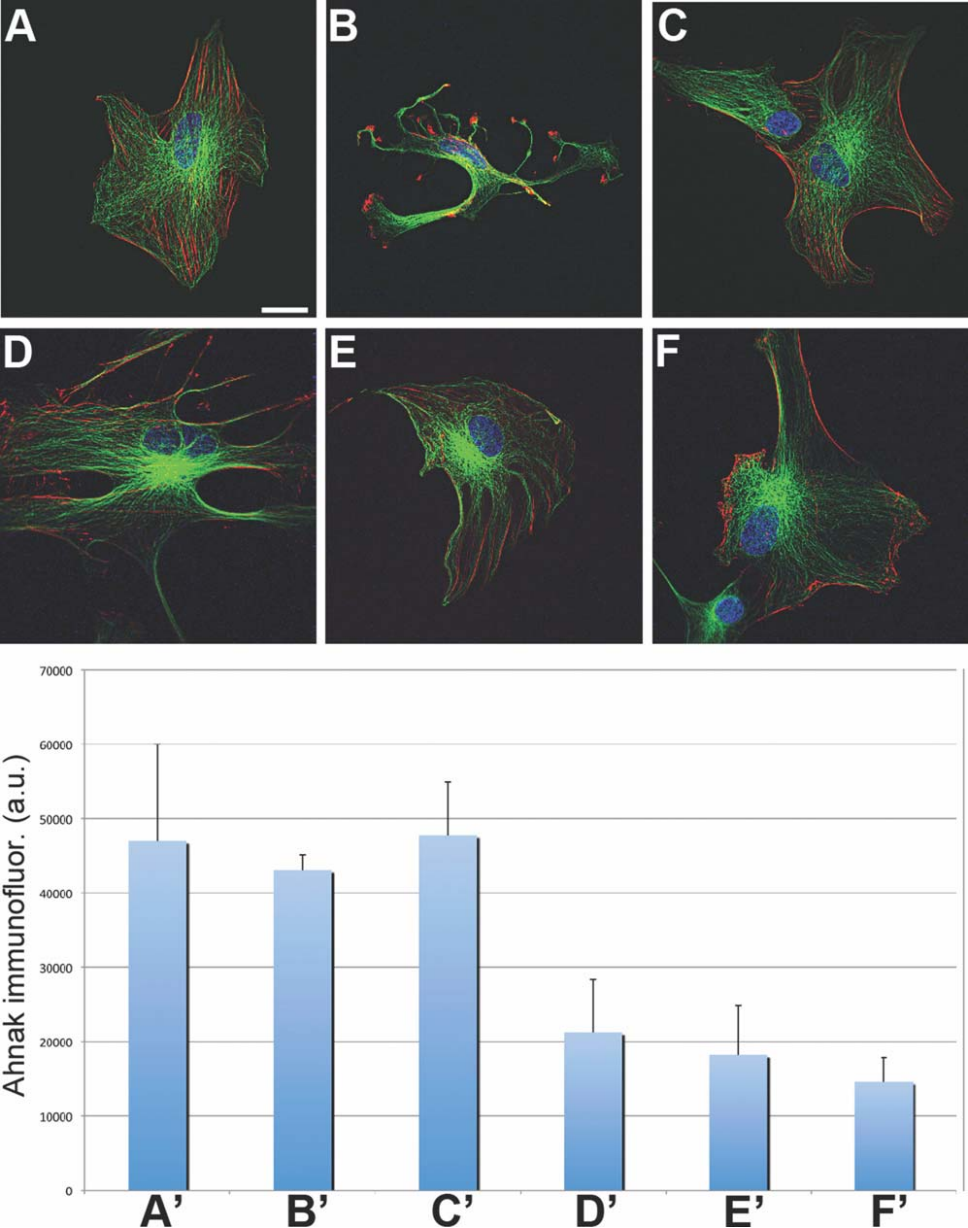
Repeated Y27632 application/washout cycles. Effects on the phenotype and Ahnak levels of cultured astrocytes. (**A**–**F**) Cultured astrocytes immuno-labeled for β-tubulin (green) and α-actin (red). (A) Before any treatment; (B) after 1 h of Y27632 (25 μM); (C) as (B) but after 2 h washout; (D) as in (C) but after 1 h reapplication of Y27632; (E) as in D but after a second 2 h washout; (F) as in (E) but after a third 1 h application of Y27632. Bar in (A), valid in (B–F), is 10 μm. (**A**′–**F**′): Levels of Ahnak assayed by quantitative immunolabeling in groups of at least 25 cells each treated as in (A–F).

ROCK blockade by Y27632 is reversible (Narumiya et al., 2000). Whether in cultured astrocytes stellation induced by the drug is dissipated on its washout and possibly re-induced by its re-application was unknown. Upon 1 h treatment with 25 μM Y27632, astrocytes were therefore exposed to various times of washout before fixation and immunolabeling for α-actin and β-tubulin. Figure 6A–C shows that 2 h washout was enough to convert the Y27632-induced stellate cells (Fig. 6B) into flat astrocytes, with disappearance of the processes, enlargement of the cell body, and reappearance of robust stress fibers (Fig. 6C). The subsequent reapplication of the drug induced outgrowth of processes, however without major reduction of the cell body volume and with no complete disappearance of stress fibers (Fig. 6D). A second washout yielded the reappearance of the flat phenotype (Fig. 6E) which was no longer modified by subsequent applications of the drug, except for an attenuation of stress fibres (Fig. 6F).

Astrocytes used in the aforementioned studies were immunolabeled in parallel for Ahnak to assay the total levels of the enlargeosome marker at the various stages of the experiment. During the initial stellation, when the marker was largely transferred to the cell surface (Fig. 5C), and during the first reflattening, after which the marker was mostly scatted in the cytoplasm (not shown), the total cell levels of Ahnak remained unchanged (Fig. 6A′-C′), suggesting the traffic of enlargeosomes by exocytosis and endocytosis to be conserved (Cocucci et al., 2007). However, during the second Y27632 treatment and in the rest of the experiment the levels decreased markedly (Fig. 6D′-F′), suggesting losses possibly due to external surface proteolysis and/or release of specific, Ahnak-enriched ectosomes (Cocucci et al., 2007). In a protocol of repeated stimulations, stellation appears therefore to undergo a progressive exhaustion which may be due, at least in part, to the exhaustion of the enlargeosome pool.

## DISCUSSION

In previous studies, stellation of flat cultured astrocytes had been induced by a variety of treatments affecting the Rho/ROCK signalling. We have used one such treatment, with the ROCK blocker Y27632. Compared to other agents, Y27632 offers several experimental advantages, i.e., stellation is dose-dependent, develops rapidly and is rapidly reversible upon washout of the drug. We found the first change induced by Y27632 to be the appearance of globular, α-actin-rich sprouts converted, within min, into processes exhibiting microtubules in their stalk and α-actin-rich cones at their tips. Similar to tissue astrocytes (Derouiche and Frotscher, 2001), those induced by Y27632 were covered by lamellipodia that exclude GFAP, rich of α-actin and of its binding protein, ezrin.

In agreement with previous studies (Favero and Mandel, 2007; John et al., 2005; Salhia et al., 2005), we confirmed stellation to depend on an extensive restructuring of the cytoskeleton, involving both microtubules and microfilaments. In fact nocodazol, that induces microtubule depolymerization, and latrunculin A, that sequesters α-actin monomers, affected stellation induced by Y27632 in different ways. The first blocked the outgrowth of processes from the α-actin-rich sprouts whereas the second altered the development of stellation inducing the assembly of discrete, α-actin-rich subplasmalemma aggregates protruding from the cell surface. Ample evidence in a variety of cell types had shown cytoskeletal reorganizations to depend on the small GTPases of the Rho family, in particular on the balance between RhoA and its effector ROCK with respect to Rac1 and cdc42 (Hall, 2005). Whether the balance of small GTPases governs also stellation, however, was still an open question. RhoA activation had been shown to induce reconversion of stellate to flat cells (Suidan et al., 1997), and inhibition of RhoA and/or of ROCK to be needed for stellation to occur (Boran and Garcia, 2007; Favero and Mandel, 2007; Hotje et al., 2005; John et al., 2004; Rosso et al., 2007). Yet, results obtained by various approaches had suggested Rac1 not to be involved (John et al., 2004; Lichtenstein etal., 2010; Rosso et al., 2007; Suidan et al., 1997; see however Salhia et al., 2005). The possible role of cdc42 had never been investigated.

In other cell types, where Rac1 is constitutively inhibited by ROCK, the changes of phenotype induced by ROCK blocker drugs, such as Y27632, are known to depend, at least in part, on the activation of Rac1 (Othta et al., 2006; Racchetti et al., 2010; Takefui et al., 2007; Yamaguchi et al., 2001). Our results demonstrate now the importance of Rac1 in astrocytes. In fact, the Y27632-induced stellation was prevented by downregulation of Rac1 and blocked by EHT 1864, a specific inhibitor of the small GTPase, and by wortmannin, an inhibitor of PI3 kinase which operates co-ordinately with Rac1 (Fukata et al., 2003). In contrast, stellation was unaffected by secramine, a blocker specific of cdc42. Taken together our results indicate Rac1, and exclude cdc42, as a key mediator of the stellation taking place in cultured astrocytes upon ROCK inhibition.

Stellation induced by Y27632 requires a huge and rapid expansion of the cell surface. Changes of this type, no matter of the cell, can only be sustained by the exocytic incorporation of intracellular membranes into the plasma membrane (Chieregatti and Meldolesi, 2005; Morris and Homann, 2001). The involvement of Rac1 in the process was not surprising because the small GTPase was already known to play stimulatory roles in various exocytic processes inhibited by RhoA, for example the release of insulin and catecholamines triggered by lipid metabolism in β-cells and chromaffin cells, respectively (Mamboisse et al., 2011; Wang and Thurmond, 2010). In these cells, however, the nature of the exocytic organelles involved had not been identified, except for their distinction from the vesicles of transmitter release (Alberts et al., 2006). Our novel finding was the identification of enlargeosomes, small vesicles competent for regulated exocytosis abundant in many cells including astrocytes (Borgonovo et al., 2002), as the organelles that participate in stellation. In addition to their rapid discharge induced by large increases of the cytosolic Ca^2+^ concentration (Borgonovo et al., 2002; Kasai et al., 1999), enlargeosomes of neural cell types had been shown to undergo slower, Ca^2+^-independent exocytoses upon Y27632 treatment, with ensuing surface enlargement and outgrowth of neurites (Racchetti et al., 2010). However, in non-neural cells such as HeLa and fibroblasts, also rich of enlargeosomes, Y27632-induced discharge occurs without significant changes of the cell shape (Racchetti et al., 2010). We have now shown that in astrocytes discharge of enlargeosomes, induced by Y27632 via the activation of Rac1, is essential for a profound phenotypic change such as stellation. Down-regulation of Rac1 prevented in fact not only stellation but also enlargeosome exocytosis. Finally, in experiments carried out according to a repeated Y27632 administration-washout protocol, stellation responses were progressively reduced and then exhausted in parallel to partial depletion of the enlargeosome pools of the cells. Taken together our results strongly suggest that, although necessary, neither the specific cytoskeleton restructuring, nor surface enlargement of the cell are sufficient to induce stellation that requires the concomitant, Rac1-dependent stimulation of both processes.

Flattening/stellation are overall phenotype changes taking place in cultured astrocytes which might be irrelevant for astrocytes in the brain. Astrocytes, however, are not static but move in the brain tissue, a function that requires cytoskeleton reorganization in discrete cell areas and membrane traffic to and from plasma membrane domains. In addition, astrocytes are able to adjust their structure/function depending on the activity of the surrounding cells. Local retractions/expansions of the astrocyte laminae ensheating various portions of neurons (Theodosis et al., 2008) modify the geometry of the extracellular space and are known to participate in multiple brain functions including synaptic plasticity (Lavialle etal., 2011; Reichembach et al., 2011). Plasticity of astrocytes themselves, often dependent on their interaction with microglia, is particularly relevant under pathological conditions (Zhang et al., 2010). Although specific studies have not been carried out yet, it is possible that mechanisms that sustain the flattening/stellation of cultured astrocytes participate also in the local astrocyte plasticity *in situ* and may therefore play significant roles in the physiology and pathology of the brain.

## Supplementary material

Supporting Information Figure 1. Spinning disc time-lapse imaging of astrocytes transiently transfected (GFP-positive, green( or not (GFP-negative) with scrambled shRNA, treated with Y27632 (25µM) at time 0. Frames recorded every 60 sec for 1 hr.

Supporting Information Figure 2. Spinning disc time-lapse imaging of astrocytes transiently transfected (GFP-positive, green) or not (GFP-negative) with the Rac1-specific shRNA, treated with Y27632 (25µM) at time 0. Frames recorded every 60 sec for 1 hr.
